# An Efficient Availability Guaranteed Deployment Scheme for IoT Service Chains over Fog-Core Cloud Networks †

**DOI:** 10.3390/s18113970

**Published:** 2018-11-15

**Authors:** Ngoc-Thanh Dinh, Younghan Kim

**Affiliations:** School of Electronic Engineering, Soongsil University, Seoul 06978, Korea; younghak@dcn.ssu.ac.kr

**Keywords:** Internet of Things, IoT service function chains, network function virtualization, fog computing, edge computing, high avaliability

## Abstract

High availability is one of the important requirements of many end-to-end services in the Internet of Things (IoT). This is a critical issue in network function virtualization (NFV) and NFV-enabled service function chaining (SFC) due to hard- and soft-ware failures. Thus, merely mapping primary VNFs is not enough to ensure high availability, especially for SFCs deployed over fog - core cloud networks due to resource limitations of fogs. As a result, additional protection schemes, like VNF redundancy deployments, are required to improve the availability of SFCs to meet predefined requirements. With limited resources of fog instances, a cost-efficient protection scheme is required. This paper proposes a cost-efficient availability guaranteed deployment scheme for IoT services over fog-core cloud networks based on measuring the improvement potential of VNFs for improving the availability of SFCs. In addition, various techniques for redundancy placement for VNFs at the fog layer are also presented. Obtained analysis and simulation results show that the proposed scheme achieves a significant improvement in terms of the cost efficiency and scalability compared to the state-of-the-art approaches.

## 1. Introduction

The Internet of Things (IoT) [1,2,3,4,5] is promising for many new applications by enabling many objects around us to connect, communicate, and interact over the Internet without human intervention. Huge data processing and data collection from sensors and other IoT devices are performed for those applications. Because IoT devices (sensors, actuators, etc.) are normally resource-constraint (i.e., storage and processing capabilities), IoT-cloud [6,7,8] was proposed as a promising approach to address the limitations. However, with a large number of devices and huge data generation, the development of IoT applications and services is a challenging task, even with the IoT-cloud architecture. Transmitting all these data to the cloud may expose excessive network bandwidth and delay. Fog/edge computing was introduced as a solution to reduce the amount of data transmitted to the cloud, improve performance, latency, and efficiency for IoT services. As a result, IoT services tend to be deployed over fog-core cloud networks. Many IoT applications such as mission-critical IoT and V2X communication require highly available services. Therefore, availability guaranteed deployment for services of those applications over fog-core cloud networks is critical.

For service deployments over cloud environments, network function virtualization (NFV) [9] is a trend. Network function virtualization (NFV) technologies are changing how network operators install and maintain their services. NFV enables operators to implement network functions (NFs) as software, known as virtual network functions (VNFs), which can be deployed on standard servers using virtual machines (VMs) or containers [10], instead of using dedicated hardware. VNFs can be used for network function deployments in both fog/edge [11,12,13] and core cloud networking [7,14].

As a typical and successful case of NFV, the service function chain (SFC) [15,16] attracts more and more attention from the industry and academia. The delivery of end-to-end IoT services often requires various network service functions. These may include traditional network service functions like firewall, load balancer, HTTP header enrichment, DPI (deep packet inspection), NAT (network address translation), etc., as well as IoT application specific functions like data aggregator, data compressor, feature extractor, or IoT gateway. Linkage of an ordered set of service functions to form a service is termed service function chaining. In IoT networks, the customers or providers can use SFC services to form a sequence of heterogeneous VNF instances for filtering, learning, using, compressing and processing the massive data flows of their applications flexibly. These SFCs can make IoT network services more efficient, scalable, and economical.

**Figure 1:**
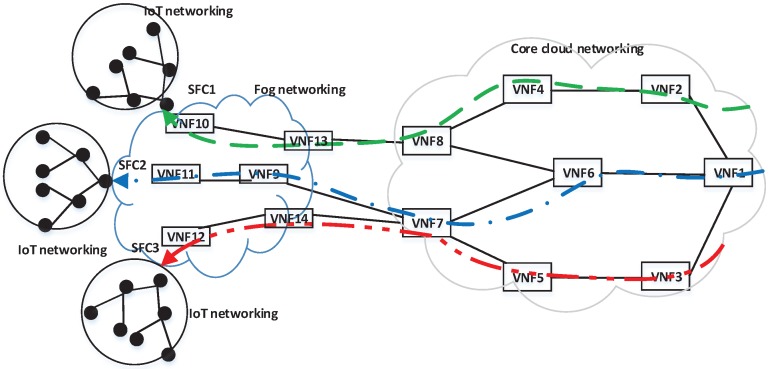
Fog-core cloud service function chains (SFCs) for IoT applications. Each SFC is deployed through a chain of virtual network functions (VNFs) across the core cloud and fog networking to serve end IoT users.

With the fog computing [17,18], several NFV-enabled network functions (i.e., virtual device driver, data aggregator, data compressor, or feature extractor, etc.) [15,16] can be deployed at the edge, so service function chains (SFCs) for various IoT services are extended across the core cloud and fog networking, as illustrated in Figure 1. Our previous implementation study [19] has shown benefits of fog/edge networking for interactive digital signage services.

However, this approach may result in vulnerabilities such as soft- and hard-ware failures. Availability/reliability is a well-known issue in NFV [20]. Once VNFs’ failures happen, especially for VNFs on fog computing domains whose infrastructure availability/reliability and resources are normally limited compared to the core cloud, the entire service function chaining (SFC) operations can be broken down. Therefore, NFV-based networks indicate higher availability requirements than conventional networks, especially for IoT services. As a result, simply embedding primary VNFs is not enough for achieving high availability and additional improvement/protection schemes are required as discussed in [20].

According to ETSI NFV architecture [9], one of the main implementation objectives of NFV is to guarantee the end-to-end availability/reliability of network services in such that not every service is required to be built to the peak. Different given resilience classes can be defined and applied depending on Service Level Agreements (SLAs). This is due to the fact that each service, or SFC, may require a different availability level depending on its demand and budget. Many applications like mission-critical Internet of Things (IoT) [8,21,22] systems, autonomous vehicular systems, and smart health-care systems require a high level of availability guarantee while web browsing services could tolerate about 30s of service interruption.

VNF redundancy deployment is one of the main protection schemes for SFCs to achieve a target service availability recommended by ETSI VNF REL 003 [20]. However, the ETSI deliverable does not provide guidance on which case and which VNFs should be selected for redundancy deployments. Applying redundancy deployment (in other words, backup) for the whole SFC is not necessary and costly as network resources are normally limited, especially in the fog computing domain. Several studies on enhancing the availability/reliability of SFCs have been introduced [23,24,25]. The current studies in this topic select the least available/reliable VNF [23] for redundancy deployment or recursively add one redundancy [24] until the availability/reliability requirement is satisfied. Those VNF redundancy allocation selections may lead to cost-inefficient protection schemes while resources at fog instances are usually limited.

According to our survey, there is currently lack of a study for availability/reliability guaranteed SFC deployment across the fog-core cloud networking. Fog computing enables to move the data transfer and processing to the edge of the cloud. However, in that environment, due to the resource limitation of fog nodes, we highlight the necessity of a cost efficient VNF redundancy deployment scheme for SFCs. According to our observation, a cost efficient VNF selection for VNF redundancy deployment depends on various factors, not just simply selecting the least reliable VNFs for redundancy deployment as studied in the recent works [23,24]. In addition, the improvement potential of VNFs in SFCs is not the same. Some VNFs require a lower cost for redundancy improvement and are more potential than others to be selected for redundancy deployment to improve the overall system availability. Initially, a cost efficient VNF selection for VNF redundancy deployment should give the priority for VNFs with a high availability improvement potential for their corresponding SFC and the low cost (i.e., CPU cost, storage cost, etc.) for redundancy deployment.

In this paper, we propose a cost-efficient availability guaranteed deployment scheme for IoT service chains over fog-core cloud networks. We first design an efficient availability-aware primary VNF embedding mechanism. We then formulate the VNF redundancy allocation cost minimization problem. For VNF redundancy allocation, we propose a metric, namely availability improvement potential per a unit cost (IPC), to measure the availability improvement potential of a VNF if the VNF is selected for redundancy allocation. The metric is used to find which VNFs are the most potential to improve, which leads to the greatest improvement to the service reliability within a limited resource required. Based on IPC, we design a cost-efficient VNF redundancy allocation scheme (IPS). Compared to the preliminary version [26], this paper provides more insight into the detailed design of the scheme with new mechanisms like the guaranteed deployment scheme for SFCs and the collaborative scheme for VNF redundancy deployment. We also present the detailed analytical model, and analyze the complexity of the scheme. We conduct more experiments and present new significant results. Analysis and Simulation results show that the proposed scheme achieves a significant enhancement in term of cost efficiency and reliability compared to REACH [23,25] and the minimum cost algorithm.

## 2. Related Work

Fog computing is proposed to extend the centralised core cloud to the edge, close proximity to the users and things in IoT [1,2,3,4,5]. The design of fog computing is to potentially improve various network services in terms of performance like reducing latency and saving network bandwidth. The fog computing pushes most of data processes out to the network edge, so several network functions are deployed at the edge. As a result, SFCs are also extended to the edge. For end-to-end SFCs over fog-core cloud networking, various technical issues are required to be solved such as task and resource allocation [27,28], service orchestration [29], fault tolerance (i.e., service reliability/availability), and management [29,30,31,32,33]. A comprehensive survey on various technical issues of fog computing can be found at [34].

With the advantages of NFV technologies, network functions including those for the edge can be implemented as software, thus facilitating the fog as well as cloud deployment [10,11,12,13,17]. Van Lingen et al. [12] argue that fog computing will become a part of convergence with NFV. Li et al. [11] design a virtual fog framework which takes the advantages of the flexibility of networking service provisioning using NFV. Ricart et al. [13] propose the TelcoFog architecture for a unified fog and cloud computing with SDN and decentralised NFV services. Richard et al. [10] implement a container-based NFV platform to facilitate the VNF deployment at the edge.

In NFV environments, guaranteeing reliability is very critical because the failure of any VNF of a particular SFC may break operations of the entire chain leading to the suspension of the network services [23,35,36,37]. In traditional network systems, all services and application are normally deployed with the same floor of availability/reliability without any distinguishment. The requirement for on-demand availability/reliability of services has not been considered in the implementation. For example, web browsing services could tolerate about 30 s of service interruption without affecting the user experience while critical IoT services are very time-sensitive and require very high availability/reliability. One of the main implementation objectives of VNF is to guarantee the end-to-end availability/reliability of network services in such that not every service is required to be built to the peak [9,20]. Different given resilience classes can be defined and applied depending on Service Level Agreements (SLAs).

A popular approach to achieve a predefined service availability/reliability is the redundancy deployment which is recommended by ETSI VNF REL 003 [20]. However, the ETSI deliverable does not provide guidance on which redundancy models should be used in which case and which VNFs should be selected for backups. Backing up the whole SFC is not necessary and costly as network resources are normally limited. In addition, we consider the VNF redundancy deployment as the cost. Therefore, optimizing the cost for VNF redundancy deployments is desirable to increase the revenue of services providers and maintain an appropriate price for network services.

Several studies on enhancing the availability/reliability in NFV environments have been introduced [23,24,25,36,38]. Long et al. [23] present an SFC deployment scheme to achieve adequate reliability guarantees for network services. In the study, the authors propose a greedy algorithm for redundant VNFs placement. The scheme first deploys primary VNFs along the chain. After the deployment of primary VNFs, if the provisioned chain has not yet met the network service reliability requirement, redundant VNFs are then deployed for the least reliable VNFs along the chain until the reliability requirement is satisfied. Similar ILP-based solutions for single-link single-node are also discussed in [38]. In GREP [36], the authors consider the whole network as a composition of several independent sub-networks. In each round, GREP selects two primary VNFs whose have the lowest reliability to provide with a backup. One backup is used for two VNFs to minimize the number of backups for a request. In [24], the authors highlight that the Internet of Things’ applications may require higher service availability for various machine control and safety-critical operations, so the availability/reliability guarantee is very important. For that purpose, a VNF deployment scheme is proposed. The scheme first embeds the primary VNF chain and then recursively deploy one redundant chain. The service availability/reliability is then updated. The scheme completes once the request reliability/availability is met or the maximum number of redundant chains is over.

According to our survey, there is currently a lack of a study for SFC deployments over the fog-core cloud networking, in which the same availability requirements are applied while the network resources are more constrained. This paper is to fulfill the gap.

## 3. The Analytical Models

This section describes the analytical model for the VNF availability used in this paper. Table 1 summarizes the parameters and variables used in this paper.

### 3.1. Network Model

We model an SFC-enabled network as a directed graph G=(N,L), where N is a set of nodes including ingress node, egress node, service nodes (SNs) including nodes in core cloud as well as in fog networking, and service function forwarders (SFFs), L represents a set of links between nodes. L(m,n) indicates the connectivity between node m and node n (i.e., L(m,n)=1). The ingress and egress node are incoming and outgoing points of flows for a given SFC. Each link is associated with a bandwidth capacity. A service node represents a cloud server or fog server that hosts virtual network functions (VNFs).

### 3.2. VNF Model

Each VNF *i* is deployed and provided several resources $R_{i}=\left\{r_{1}^{i},r_{2}^{i},\ldots ,r_{m}^{i}\right\}$ such as the number of CPU cores, storage, … VNF instances can be created with various virtual machine (VM) sizes depending on the incoming traffic flow rates. VNF instances are implemented with specific functions (i.e., firewall, proxy, video/image optimizer, data aggregator …) and embedded into nodes on the core cloud or at the edge. A VNF may serve for several SFCs. For example, a video/image optimizer or data aggregator at the edge or firewall on the core cloud may serve various services simultaneously. Each VNF type *i* is deployed on a server *e* having a reliability $r_{i}$ and availability $a_{i}$. Assume that we have a set of VNF types $F=\{f_{1},f_{2},\ldots ,f_{X}\}$. Intuitively, the amount of incoming flows to a VNF instance or a link cannot exceed their capacity.

### 3.3. Service Function Chaining Model

We consider a set S of SFC requests, $S=\{s_{k}|k=1,2,3,4,\ldots ,K\}$. Each SFC $s_{k}$ consists of m VNFs in order $s_{k}=\left\{f_{1}^{k},f_{2}^{k},\ldots ,f_{m}^{k}\right\}$. Please note that an SFC represents an order set of VNFs which are deployed for some services. As a result, traffic flows of an SFC $s_{k}$ are processed in the VNF order vector $O_{s_{k}}=\left\{f_{1}^{k},f_{2}^{k},\ldots ,f_{m}^{k}\right\}$. Each SFC *s* may have a different QoS requirement (i.e., availability requirement $R_{s}^{r}$). To deploy an SFC, a provider has to plan a right placement of VNFs and chain them through VNF forwarding graph embedding so that the end-to-end SFC’s availability satisfies the given requirement. Several SFCs may share a VNF instance as long as the VNF and links have enough capacities.

### 3.4. Availability/Reliability Model

This section presents the availability/reliability model for a given SFC. We assume the availability/reliability of service functions is configured independently, so the failure of VNFs happens independently. The assumption is the same as stated in ETSI GS NFV REL 003 [20]. The definitions of VNF availability/reliability are given in [20]. Please note that availability and reliability can be used interchangeably in this paper.

The availability and reliability of a complex composed system like SFC deployment can be modeled by disintegrating it down to its subcomponents like VNFs, of which the availability and reliability are known. There are two basic forms of combination, parallel and serial. In this paper, we use serial dependence mainly as all SFCs can be transformed into serial dependency. A serial dependency of two VNFs indicates that both are required to operate in order for the SFC to operate. Therefore, the availability of an SFC consisting of M serial VNFs is as follows.

(1) $$R_{s_{k}}\left(t\right)=\prod_{f_{m}\in s_{k}}A_{f_{m}}\left(t\right)$$

VNF redundancy deployments normally use a parallel dependency to improve the availability/reliability. In that case, the availability of a subcomponent consisting of 2 parallel dependent VNFs, VNF1 and VNF2, is calculated as follows.

(2) $$R_{sub}=1-\left(1-A_{VNF1}\right)\left(1-A_{VNF2}\right)$$

### 3.5. Cost Model

#### 3.5.1. Capital Expenditure (CAPEX)

We denote $C_{capex}^{i}$ as the deployment cost of an SFC $s_{k}$ in term of a resource type *i*

(3) $$C_{capex}^{i}=\sum_{s_{k}\in S}\sum_{f_{m}\in s_{k}}p_{m}^{k}r_{i}$$

where $p_{m}^{k}$ is the number of VNF instances of type $f_{m}^{k}$ used.

The deployment cost can be the financial cost (i.e., license cost for VNFs), processing (i.e., the number of CPUs), or storage cost. In this paper, we use the number of CPUs as the deployment cost.

#### 3.5.2. Operating Expenditure (OPEX)

We denote $C_{opex}$ as the operating cost of an SFC $s_{k}$.

(4) $$C_{opex}=\sum_{f_{m}\in s_{k}}\sum_{i=1}^{p_{m}^{k}}E_{f_{m}}$$

where $E_{f_{m}}$ is the operating cost consumption rate of an instance of VNF type $f_{m}$. The operating cost consumption can be the energy cost, bandwidth cost, or even management cost.

## 4. An Efficient Availability-Aware Primary VNF Embedding Mechanism

We assume there is several SFCs for deployments. The task is to deploy the SFCs to meet their predefined availability requirement with a cost efficiency. For a given SFC request $s_{k}$, we first need to deploy the primary VNFs before redundancy deployments are performed. For the above objective, we design an efficient availability-aware primary VNF embedding mechanism as follows.

We call $H_{ij}$ as the hop count distance between VNF *i* and VNF *j*, where *i* is a current VNF in the chain $s_{k}$ and *j* is a candidate for next hop of *i* in the chain $s_{k}$. The availability score of *j* is $R_{j}$ and the availability-cost ratio (RCR) $r_{ij}^{c}$ for *i* to select *j* as the next hop is defined as follows.

(5) $$r_{ij}^{c}=\frac{R_{j}}{H_{ij}}$$

The detailed algorithm for the primary VNF embedding is presented in Algorithm 1. For an SFC $s_{k}$ consists of M VNF types in order $s_{k}=\left\{f_{1}^{k},f_{2}^{k},\ldots ,f_{m}^{k}\right\}$, the embedding scheme for the primary VNFs starts from $f_{1}^{k}$. If there are *N* VNF instances of $f_{1}^{k}$ are available and have enough capacity for $s_{k}$, the VNF instance with the greatest value of the availability-cost ratio $r_{ij}^{c}$ is selected as a primary VNF for deploying $s_{k}$. The purpose is to maximize the availability of $s_{k}$ within a limited cost (i.e., bandwidth).

If there is no available VNF instance of $f_{1}^{k}$ having enough capacity for $s_{k}$, a new VNF instance of $f_{1}^{k}$ needs to be instantiated. The new VNF deployment policy is as follows. The new VNF instance is deployed as close as possible to the previous VNF to minimize the bandwidth consumption. This proximity-based policy is also aligned with the deployment strategy of fog instances to save the network resources and improve the performance. The procedures are executed repeatedly until all of the primary VNFs of the chain $s_{k}$ are deployed.

After all of the primary VNFs are deployed, an availability score check is executed for $s_{k}$. If the requested availability requirement of $s_{k}$ is satisfied, the mechanism stops and the SFC deployment is completed. Otherwise, the below redundancy allocation scheme is called.

**Algorithm 1** Primary VNF Embedding Scheme**INPUT:**G(N,L), A set of SFC requests $S=\{s_{k}|k=1,2,3,4,\ldots ,K\}$, $s_{k}=\left\{f_{1}^{k},f_{2}^{k},\ldots ,f_{m}^{k}\right\}$ **OUTPUT:** The primary VNF embedding plan
**Initialize:** Calculate $r_{ij}^{c}$ for related VNF instance *j*
**Repeat**
  **for all**
$s_{k}\in S$
**do**
    **for**
i=1;i≤m;i++
**do**
      **if**
$AvailableVNFInstances(f_{i}^{k})\geq 1$
**then**
        SelectMaxRCR(AvailableVNFInstances($f_{i}^{k}$));
      **else**         ProximityBasedNewVNFDeployment($f_{i}^{k}$);
      **end if** 
    **end for**
    $R_{s_{k}}$ = AvailabilityScoreCheck($s_{k}$);
    **if**
$R_{s_{k}}\geq R_{s_{k}}^{requirement}$
**then**
      Complete();
    **else**
      IPS-RedudancyAllocation();
    **end if**
  **end for**
  **UNTIL**
$\forall s_{k}$, $s_{k}$ is embedded or resources run out.

## 5. The VNF Redundancy Allocation Cost Minimization Problem

As the primary VNF deployments of SFCs is normally not enough to satisfy the availability requirements of services, VNF redundancy deployments are thus required. In this section, we define a VNF redundancy cost minimization problem to meet a predefined availability and formulate the problem using an Integer Linear Programming (ILP) model as follows.

### 5.1. Objective Function

Given a set S of SFCs consisting of primary VNFs deployed in the network, we find the optimal VNF redundancy deployment so that

- Availability requirements of the SFCs are satisfied
- The redundancy deployment cost (i.e., the number of CPUs) is minimized.

We define a decision binary variable $b_{f_{i}^{k}s_{k}}^{p}$. $b_{f_{i}^{k}s_{k}}^{p}=1$ if the primary VNF $f_{i}^{k}$ of SFC $s_{k}$ on the physical server *p* is selected for redundancy deployment. Otherwise, $b_{f_{i}^{k}s_{k}}^{p}=0$. We call $C_{f_{i}^{k}s_{k}}$, $C^{c}p_{f_{i}^{k}s_{k}}$, and $C_{f_{i}^{k}s_{k}}^{m}$ as the general cost, the compute cost, and the storage cost for a redundancy deployment, respectively. $B_{f_{i}^{k}s_{k}}^{r}$ is the required bandwidth of service $s_{k}$ at VNF $f^{k}$. We assume $C_{f_{i}^{k}s_{k}}$ is equal to the resource required by the primary VNF $f_{i}^{k}$ and a redundancy of $f_{i}^{k}$ has the same availability of $f_{i}^{k}$. The objective of the ILP model is to minimize the redundancy deployment cost. Mathematically, this objective is given as follows.

(6) $$Minimize\sum_{s_{k}\in S}\sum_{f_{i}^{k}\in s_{k}}b_{f_{i}^{k}s_{k}}^{p}C_{f_{i}^{k}s_{k}}$$

### 5.2. Constraints

The total used capacity of VNFs hosted by a physical server p should be equal to or smaller than the total compute capacity of p ($C_{p}^{c}$).

(7) $$\sum_{s_{k}\in S}\sum_{f_{i}^{k}\in p_{v}}b_{f_{i}^{k}s_{k}}^{p}C_{f_{i}^{k}s_{k}}^{cp}\leq C_{p}^{c}$$

where $p_{v}$ is the set of VNFs hosted by a physical server *p*.

Similarly, the total used memory capacity of VNFs hosted by the physical server *p* should be equal to or smaller than the total memory capacity of p ($M_{p}^{c}$)

(8) $$\sum_{s_{k}\in S}\sum_{f_{i}^{k}\in p_{v}}b_{f_{i}^{k}s_{k}}^{p}C_{f_{i}^{k}s_{k}}^{m}\leq M_{p}^{c}$$

where $p_{v}$ is the set of VNFs hosted by a physical server *p*. Required bandwidth capacity of a set $l_{v}$ of VNFs mapped using a substrate link *l* must be equal or less than the link capacity of *l* ($B_{l}^{c}$)

(9) $$\sum_{s_{k}\in S}\sum_{f_{i}^{k}\in l_{v}}B_{f_{i}^{k}s_{k}}^{r}\leq B_{l}^{c}$$

The total required processing resources of a set $S^{i}$ of SFCs that pass a VNF $f_{i}$ should not exceed the processing capacity of the VNF $f_{i}$.

(10) $$\sum_{s_{k}\in S^{i}}C_{s_{k}}^{cp}\leq C_{f_{i}}^{c}$$

This means that a new SFC can be embedded through a shared VNF *i* if and only if the VNF *i* has enough capacity for processing.

(11) $$0\leq R_{i}\leq 1\left(\forall VNFi\right)$$

$b_{ij}$ is the binary variable, so we have the following constraint.

(12) $$b_{ij}\in \left\{0,1\right\}$$

## 6. A Cost-Efficient Redundancy Allocation Scheme for VNFs

### 6.1. Reliability Importance Measure

In availability/reliability theory [39], the component availability importance measures are used widely in availability/reliability optimization to focus on enhancements with the greatest reliability improvement. Among them, Birnbaum Importance Measure (BIM) and Improvement Potential Measure (IPM) [39] are the popular indexes. BIM measures the importance of a component’s availability/reliability in a system. PIM measures the improvement potential of the system availability/reliability if the component *i* is replaced by a perfect component or is upgraded (i.e., deploy a redundancy for *i*).

### 6.2. A Cost-Efficient Improvement Potential Measure for VNFs

In availability/reliability theory [39], the improvement potential is used to represent the maximum potential improvement in the system availability/reliability that can be obtained by improving the availability/reliability of component *i*. The availability of a component *i* may be improved by using a higher quality component, deploying redundant components, decreasing operating loads, or improving the maintainability of the component. We find that the above features make the improvement potential a good candidate to measure the availability/reliability improvement potential of VNFs in SFCs. The improvement potential of a VNF *i* is defined as follows.

(13) $$I^{IP}\left(i\right)=h\left(p_{i},p\left(t\right)\right)-h\left(p\left(t\right)\right)$$

where $h(p_{i},p\left(t\right))$ is the availability of the system when the availability of *i* is upgraded to $p_{i}$ (i.e., $p_{i}$ can be the maximum availability of *i* or a new availability value) and h(p(t)) is the availability of the system with the current VNF *i*. According to the availability/reliability theory, the cost to achieve $p_{i}$ is $C^{p_{i}}$. The theory in [39] proves that selecting the component with the greatest improvement potential leads to the maximum improvement to the system availability.

However, in practice, it is difficult to achieve the maximum availability of *i* and the cost specification to achieve a new availability $p_{i}$ value of *i* may be not available. Moreover, the availability improvement for an SFC is normally executed within a limited budget and resource. The cost $C^{p_{i}}$ may be un-affordable. For a realistic improvement potential computation, we adapt $p_{i}$ as the new availability of *i* after a parallel redundancy of *i* is added. We then establish a metric, namely the improvement potential per a unit cost ($I^{IPC}\left(i\right)$), to evaluate the availability improvement potential can be achieved within a unit cost for the redundancy deployment of VNF *i*. $I^{IPC}\left(i\right)$ is calculated as follows.

(14) $$I^{IPC}\left(i\right)=\frac{h\left(p_{i},p\left(t\right)\right)-h\left(p\left(t\right)\right)}{C^{p_{i}}}$$

$I^{IPC}\left(i\right)$ is used to make an efficient and feasible protection scheme for VNFs in SFCs.

### 6.3. A Cost Efficient VNF Redundancy Allocation Scheme

Based on $I^{IPC}\left(i\right)$, we design an efficient improvement potential-based VNF redundancy allocation scheme (IPS) for the availability improvement of SFCs.

The proposed VNF redundancy allocation scheme (IPS) is presented in Algorithm 2. For the SFC $s_{k}$ consists of M VNF types in order $s_{k}=\left\{f_{1}^{k},f_{2}^{k},\ldots ,f_{m}^{k}\right\}$, IPS calculates $I^{IPC}\left(i\right)$ for each VNF $f_{i}^{k}$. The VNF with the greatest value of $I^{IPC}\left(i\right)$ is selected for redundancy deployment first, which leads to the greatest availability improvement to the system within a limited cost. The VNF redundancy placement on the core cloud simply follows the guidance in ETSI VNF REL [20] in which the redundant VNF should be placed at a nearby physical server. Due to the resource limitation, we design a collaborative VNF redundancy placement scheme for VNFs at the fog layer, as presented in the next section. After a redundancy deployment, the scheme re-calculates the availability score for $s_{k}$. If the availability requirement of $s_{k}$ is satisfied, the VNF redundancy deployment scheme stops. Otherwise, the VNF redundancy deployment is executed repeatedly until the requirement is satisfied or the resources run out.

**Algorithm 2** IPS Algorithm for VNF Redundancy Allocation**INPUT:**G(N,L), A set of SFCs $S=\{s_{k}|k=1,2,3,4,\ldots ,K\}$ where $R_{s_{k}}<R_{s_{k}}^{requirement}$, $s_{k}=\left\{f_{1}^{k},f_{2}^{k},\ldots ,f_{m}^{k}\right\}$
**OUTPUT:** The VNF redundancy allocation embedding plan
**Initialize:** Calculate $I^{IPC}\left(f_{i}^{k}\right)$, $f_{i}^{k}\in s_{k}\in S$
**Repeat**
  **for all**
$s_{k}\in S$
**do**
    **while**
$R_{s_{k}}<R_{s_{k}}^{requirement}$
**do**
      MaxIPSBasedVNFRedundancyAllocation($s_{k}$);
      $R_{s_{k}}$ = ReliabilityCheck($s_{k}$);
    **end while**
  **end for**
  **UNTIL**
$\forall s_{k}$, $R_{s_{k}}\geq R_{s_{k}}^{requirement}$ or resources run out.

The objective of the scheme is that within a limited given resource, the scheme selects the VNF of $s_{k}$ for a VNF redundancy deployment which leads to the greatest improvement per a unit cost in the availability of the system (i.e., SFC). For example, among VNFs of a service shown in Figure 1, the VNF 9 has a significant improvement potential (i.e., 0.12) with a corresponding low cost (i.e., 2 CPUs) which leads to the greatest improvement potential per a unit cost. The selection scheme prioritizes to allocate a redundancy for VNF 9 to improve the availability of the corresponding SFC.

### 6.4. A Collaborative Redundancy Placement Scheme for VNFs at the Fog Layer

**Figure 2:**
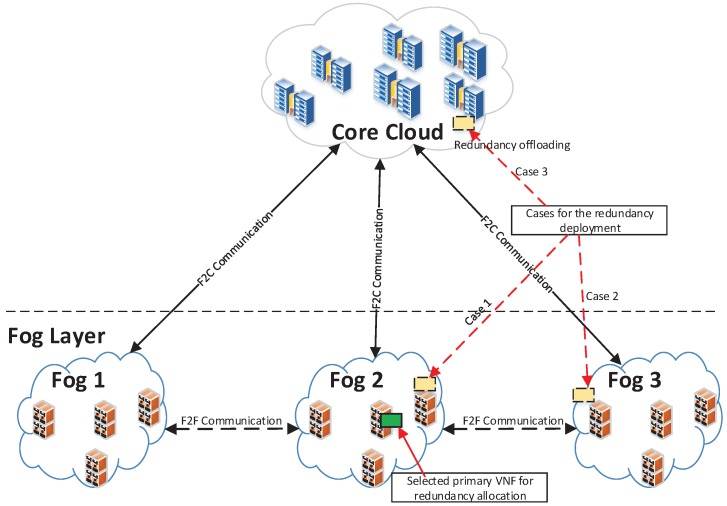
Scenarios for the redundancy deployment of the primary VNF at the fog layer.

In fog computing, the fog layer consists of several fogs and each fog as a micro-datacenter may consist of several micro-servers with different levels of storage and processing capacity for various services. They normally have limited storage and processing capacity compared to the core cloud, so a fog may not always have enough resources for a VNF redundancy placement. For that reason, we design a collaborative redundancy placement scheme for VNFs at the fog layer, which exploits the collaboration between a fog with other fogs, and between a fog with the core cloud. The scheme is presented in Algorithm 3 and illustrated in Figure 2.

The scheme works as follows. We assume that a primary VNF *f* in a node $n_{p}$ of a fog *O* is selected for a redundancy allocation. Three scenarios for VNF redundancy placements are shown in Figure 2. Fogs are connected with each other through fog-to-fog (F2F) communication links and with the core cloud through fog-to-cloud (F2C) communication links. The scheme first looks for deploying a redundancy of *f* in a nearby physical server in the same fog to optimize the latency while the availability improvement is secured. If the current fog *O* does not have enough resources for the redundancy, the scheme searches for a resource allocation at nearby fogs. As nodes in a fog can be organized hierarchically, the scheme considers only nodes in the same fog level and upper levels. The reason is that those nodes normally have equal or richer resources than the current node that contains the primary VNF. We assume there are *e* available nodes, $E=n_{1},n_{2},\ldots ,e_{n}$, in nearby fogs having enough resources and are able to accept the redundancy placement. Their corresponding round-trip latency values from the $n_{p}$ are $l_{1},l_{2},\ldots ,l_{e}$. The scheme selects the node $n_{select}$ with the lowest latency $l_{min}=min\left\{l_{1},l_{2},\ldots ,l_{e}\right\}$ for the redundancy placement if $l_{min}$ is lower than the latency from the node to the core cloud $l_{core}$.

If there is no available node in nearby fogs be able to accept the redundancy placement or the latency to available nodes is greater than $l_{core}$, the scheme makes a decision to off-load the VNF redundancy to the core cloud. The node with the lowest latency on the core cloud is selected for the VNF redundancy placement.

The decision for the redundancy placement scenarios can be made in a distributed manner by negotiation among nodes or in a centralised manner. In the case of the centralised approach, according to the architecture of NFV and Software Defined Networking (SDN) [40,41], this kind of orchestration decision should be implemented at the orchestrator and supported by the controller.

**Algorithm 3** A Collaborative Redundancy Deployment Scheme for VNFs at the Fog Layer**INPUT:**G(N,L), a primary VNF *f*, its redundancy cost *c*, node *P*,$P_{neighbor-nodes}$, fog *O*, $O_{neighbor-fogs}$ **OUTPUT:** The selected node for deploying the redundancy of *f*
**Initialize:** Selected node s=0, $l_{min}=\infty$, $l_{core}$
  **for all**
$i\in P_{neighbor-nodes}$
**do**
    **if**
i.Available(f,c)==1
**then**
      **if**
$l_{i}<$ & $l_{i}<l_{core}$
**then**
        s=i;
        $l_{min}=l_{i}$;
      **end if**
    **end if**
  **end for**
  **if**
s==0
**then**
    s=Neighbor_Fog_Search(f,c);
  **end if**
  **if**
s==0
**then**
    s=Redundancy_Cloud_Offloading(f,c);
  **end if**
  **RETURN**
*s*

**Redundancy offloading:** Due to the resource limitation, when resources at the fog layer are not enough for a redundancy deployment, the redundancy offloading to the core cloud is used. However, there is a difference in the usage between a normal redundant VNF (i.e., in the same fog layer with the primary VNF) and an offloading redundant VNF. While a normal redundant VNF is designed to replace the role of the primary VNF when the primary VNF fails, an offloading redundant VNF is only used temporarily to operate the network function during the failed period of its primary VNF. When the primary VNF is repaired, the role is returned to the primary VNF and the offloading redundant VNF also returns to its backup role. This is to enhance the availability when the primary VNF fails temporarily. The role returning is to exploit the advantages of fog computing.

## 7. Performance Evaluation

This section presents the performance evaluation for the proposed scheme in comparison with REACH [23,25], the state-of-the-art VNF redundancy deployment scheme, and MC, the scheme which selects the VNF with the minimum resource requirement for redundancy deployment. As REACH and current schemes do not distinguish fog nodes and core cloud nodes, to make results compatible, we consider the whole fog and core-cloud networks in a single network graph and provide the general results.

We use CPLEX solver to solve the ILP model and analysis which are also used in REACH [23,25]. The simulations [42] and analysis are run on a PC equipped with an Intel 3.5 GHz and 10 GB RAM. All analysis and simulations are performed with a network composed of 40 physical servers for the core-cloud and 4 fogs with 4 physical nodes for each. Each server can provide three types of resources, namely CPU, memory, and storage, with a capacity of 20 to 100 units for each type of resource. We assume the resources of a fog node equal to one-third of a cloud server. Each SFC requests from 4 to 8 VNFs. We assume there are 20 types of VNFs for core-cloud and 5 types of VNFs for fog networking. Each type of VNFs requires the three types of resources. The VNF demand for each type of resource is distributed between 1 and 8. Similar to REACH, SFCs are composed randomly and the links are assumed to have a perfect reliability.

The reliability of each VNF is randomly distributed within 0.9 and 0.99. Each SFC request has the reliability requirement among 95%, 99%, 99.9%, 99.95%, following the configuration in the previous studies [23,25]. The link rates between fog nodes in one domain are 100 Mbps and that of the path from fog nodes to cloud servers are 10 Gbps. For a fair comparison, other parameters are similar to those used in REACH [23,25]. We reuse the theoretical modeling and setting for the fog layer presented in [43]. Our previous implementation study [19] also illustrates the benefits of fog/edge networking and processing operations between the edge and the core cloud.

### 7.1. Complexity Analysis

In this subsection, we discuss the complexity analysis for the VNF placement algorithm. It is obvious that the proposed VNF placement algorithm is a heuristic iterative-based algorithm. It has a similar complexity compared to REACH [23,25]. The generation of an SFC path has a complexity of $O(|N|(|F_{i}\left|+|M|+|N|)\right)$ = $O(N^{2})$ ( $|F_{i}\left|\leq |N\right|$ and |M|≤|N|), where $|F_{i}|$ is a set of VNF instances of the network service *i* and *M* is the length of the path. The worse case of VNF placements has a complexity of $O(N^{2})$.

### 7.2. IPS and ILP

This subsection compares the performance as well as the overhead of IPS and ILP. Results are presented in Table 2.

The results show that both the ILP and IPS achieve the availability requirement of the network services. The ILP solution requires a much longer time to solve the problem. For example, ILP needs 9425 s to solve the problem for the requirement of 0.95 and 26,263 for the requirement of 0.9995, even for this small network. On the other hand, IPS is able to find the solutions within only 0.72 s while the cost efficiency of IPS is only slightly lower than the ILP. The results are due to the fact that the ILP solution resorts to solve the ILP model at each iteration of the algorithm. The results clearly indicate that IPS achieves much better scalability than the ILP while the ILP may be not appropriate to use in operations due to time complexity. Therefore, we focus on evaluating the performance of IPS in the remaining of this section.

### 7.3. CPU Unit Cost for Redundancy Deployment

**Figure 3:**
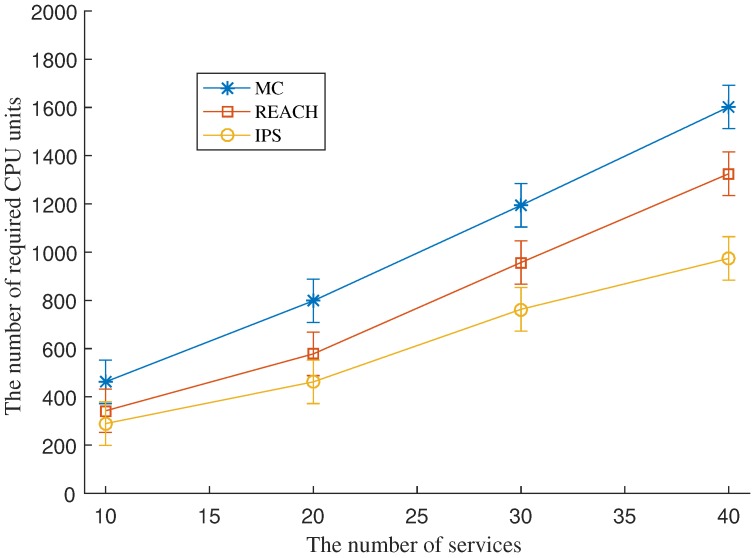
The number of required CPU units for redundancy deployment of MC, REACH, and IPS.

We set a fixed service availability requirement to 0.99 and vary the number of SFC requests. The schemes select VNFs for redundancy deployment until the service availability requirement of SFCs is satisfied. Figure 3 shows the number of CPU units required by each scheme under a various number of SFC requests. The figure shows that IPS consumes fewer CPU resources for the same number of SFC requests, compared to REACH and MC. The higher the number of SFC requests the better the cost efficiency IPS can achieve compared to REACH. MC is the most expensive mechanism among the three.

### 7.4. Scalability Test

**Figure 4:**
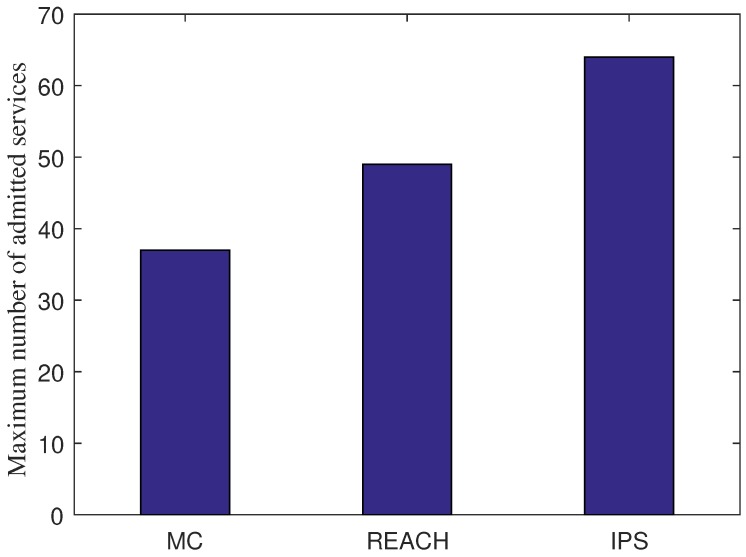
The maximum number of admitted services each scheme can afford.

We perform a scalability test by adding more SFC requests until there is not enough resource for more SFC deployment. Through the scalability test, we find the maximum number of admitted services that each scheme can afford, as shown in Figure 4. It is obvious that IPS achieves the better scalability by allowing more services can be admitted (i.e., 64 services) compared to 49 services in the case of REACH and 37 services in the case of MC. This figure implicates that by saving the redundancy deployment resource, the service providers can deploy more services within a limited amount of resource. As a result, IPS can help increase the revenue of the service providers.

### 7.5. Under Service Availability Requirement Variation

**Figure 5:**
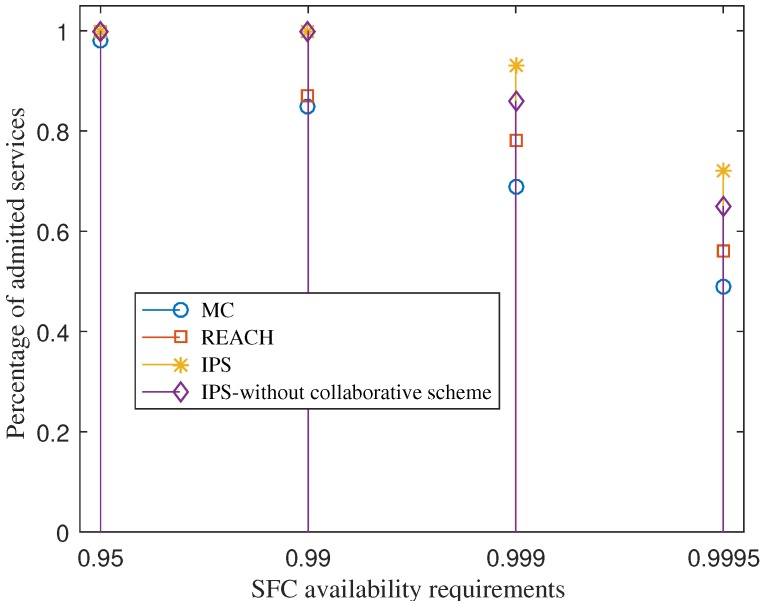
The percentage of admitted services under various service reliability requirements.

We now deploy totally 60 SFC requests and vary the availability requirement of services. This experiment is to study the performance behavior of each scheme in the case of fixed network resources under various service requirements. Figure 5 shows the percentage of admitted services when the service availability requirements increase. The figure indicates that within a limited resource, the higher the service availability requirement the lower the number of SFC requests can be admitted. The reason is that more redundancy deployment is required for each service. By considering the availability improvement potential of VNFs and its redundancy cost seriously, IPS can enable a greater number of services that are admitted, compared to REACH and MC. This helps increase the revenue of service providers.

The figure also shows that IPS with the collaboration scheme achieves a greater percentage of admitted services than IPS without collaborative under the SFC availability requirements of 0.999 and 0.9995. Both of the schemes achieve the same percentage of admitted services under the SFC availability requirements of 0.95 and 0.99. The reason is that under the requirements of 0.95 and 0.99, IPS can admit all the SFC requests without requiring offloading VNF redundancies. The fog layers have enough resources for the SFC deployments. At the higher requirements of 0.999 and 0.9995, each SFC requires a greater number of VNF redundancy deployments, so resources at the fog layer maybe not enough for all services. By using the collaborative scheme, IPS can increase the percentage of admitted services, compared to IPS without the collaborative scheme.

**Figure 6:**
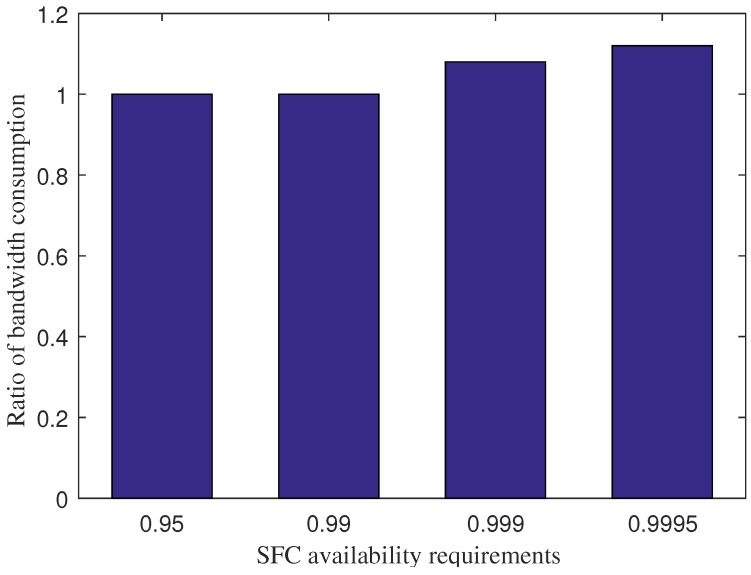
The ratio between of the bandwidth consumption of IPS and IPS without the collaborative scheme under various SFC availability requirements.

The trade-off of IPS for increasing the percentage of admitted services is the higher bandwidth consumption for information exchange during the VNF redundancy deployment, as shown in Figure 6. Under the requirements of 0.95 and 0.99, IPS and IPS without the collaborative scheme consume the same amount of bandwidth. However, the bandwidth consumption of IPS is higher than IPS with the collaborative scheme by 8 % and 12 % under the requirements of 0.999 and 0.9995, respectively. The reason is that IPS performs offloading several VNF redundancies for several primary VNFs at the fog layer to the core cloud at a longer distance. The trade-off is appropriate as the bandwidth consumption is relatively small during the VNF redundancy deployment and for the cases with a high availability requirement only.

## 8. Discussion and Conclusions

This paper applies the availability/reliability theory to VNF protection scenarios to enhance the availability/reliability improvement of SFCs over fog-core cloud networking, in which network resources are limited. We propose a metric to measure the improvement potential per a unit cost of VNFs for redundancy deployments. Based on that we design an efficient redundancy deployment scheme and a primary VNF deployment scheme for VNFs to meet predefined reliability requirements. Obtained simulation and analysis results show that the proposed scheme achieves a significant improvement in term of cost efficiency and scalability compared to the current approaches. The results also show the advantages of the collaborative scheme for the VNF redundancy deployment which increases the percentage of admitted services in fog - core cloud environments. Our analysis indicates that the bandwidth consumption is a tradeoff of the collaborative scheme. Through discussions, we highlight the tradeoff is appropriate. For future works, we plan to investigate other requirements (i.e., the latency) for SFC deployments over the fog-core cloud. A latency guaranteed deployment for SFCs is also an issue in the fog-core cloud environments, especially for IoT services. The issue becomes more complicated when we consider the involvement of low duty cycled wireless sensor networks for those IoT services. We also plan to extend the model to support VNF migrations and mobility scenarios. In such scenarios, the adaptability of SFCs is required because the demand of each network function is dynamically changed regarding to the mobile users. The availability protection plan is also critical to the network slicing. However, unique characteristics of the network slicing should be addressed. Therefore, we also plan to modify the model to support the network slicing.

## Figures and Tables

**Table 1:** Parameters and variables.

Parameter	Meaning
N	a set of nodes
L	a set of links between nodes
$r_{i}$	reliability of *i*
$a_{i}$	availability of *i*
$S=\{s_{k}|k=1,2,3,4,\ldots ,K\}$	a set S of SFC requests
$F=\{f_{1},f_{2},\ldots ,f_{X}\}$	a set of VNF types
$s_{k}=\left\{f_{1}^{k},f_{2}^{k},\ldots ,f_{m}^{k}\right\}$	M VNFs in order of an SFC $s_{k}$
$R_{s}^{r}$	reliability requirement of SFC *s*
$R_{s_{k}}$	reliability of an SFC $s_{k}$
$A_{s_{k}}$	availability of an SFC $s_{k}$
$C_{capex}^{i}$	reliability of VNF *j*
$r_{i}$	capital expenditure
$C_{opex}$	operating expenditure
$p_{m}^{k}$	the number of VNF instances of type $f_{m}^{k}$
$t_{m-p}^{k}$	the data rate of the $p^{th}$ instance of VNF $f_{m}^{k}$
$E_{f_{m}}$	energy consumption rate of VNF type $f_{m}$
$b_{f_{i}^{k}s_{k}}^{p}$	a decision binary variable
$C_{f_{i}^{k}s_{k}}$	general cost for redundancy deployment of VNF $f_{i}^{k}$
$C^{c}p_{f_{i}^{k}s_{k}}$	compute cost for redundancy deployment of VNF $f_{i}^{k}$
$C_{f_{i}^{k}s_{k}}^{m}$	memory cost for redundancy deployment of VNF $f_{i}^{k}$
$M_{p}^{c}$	total memory capacity of *p*
$C_{p}^{c}$	total compute capacity of *p*
$B_{l}^{c}$	link capacity of link *l*
$r_{ij}^{c}$	reliability-cost ratio for *i* to select *j* as the next node
$I_{f}^{B}\left(i\right)$	Birnbaum Importance Measure of VNF *i* in *f*
$h(p_{i},p\left(t\right))$	reliability of the system when a redundancy of VNF *i* is deployed
$h(p_{i}^{max},p\left(t\right))$	reliability of the system when the maximum reliability of VNF *i* can be achieved
h(p(t))	The current reliability of the system
$C^{p_{i}}$	cost to achieve $p_{i}$
$I^{IPC}\left(i\right)$	Improvement potential per a unit cost of *i*

**Table 2:** A comparison of performance and the computation time overhead of ILP and IPS.

Scheme	Availability Requirement	Achieved Availability	Computation Time
ILP	0.95	0.9504	9425
	0.99	0.9918	17,341
	0.999	0.99908	24,152
	0.9995	0.999515	26,263
IPS	0.95	0.9517	0.36
	0.99	0.9925	0.45
	0.999	0.99923	0.61
	0.9995	0.99953	0.72
